# Sensor Life and Overnight Closed Loop: A Randomized Clinical Trial

**DOI:** 10.1177/1932296816678631

**Published:** 2016-11-11

**Authors:** Martin Tauschmann, Janet M. Allen, Malgorzata E. Wilinska, Yue Ruan, Hood Thabit, Carlo L. Acerini, David B. Dunger, Roman Hovorka

**Affiliations:** 1 Wellcome Trust-MRC Institute of Metabolic Science, University of Cambridge, Cambridge, UK; 2 Department of Paediatrics, University of Cambridge, Cambridge, UK

**Keywords:** closed loop, artificial pancreas, continuous glucose monitoring, sensor accuracy, sensor life

## Abstract

**Background:**

Closed-loop (CL) systems direct insulin delivery based on continuous glucose monitor (CGM) sensor values. CGM accuracy varies with sensor life, being least accurate on day 1 of sensor insertion. We evaluated the effect of sensor life (enhanced Enlite, Medtronic MiniMed, Northridge, CA) on overnight CL.

**Methods:**

In an open-label, randomized, 2-period, inpatient crossover pilot study, 12 adolescents on insulin pump (age 16.7 ± 1.9 years; HbA1c 66 ± 10 mmol/mol) attended a clinical research facility on 2 overnight occasions. In random order, participants received CL on day 1 or on day 3-4 after sensor insertion. During both periods, glucose was automatically controlled by a model predictive control algorithm informed by sensor glucose. Plasma glucose was measured every 30 to 60 min.

**Results:**

During overnight CL (22:30 to 07:30), the proportion of time with plasma glucose readings in the target range (3.9-8.0 mmol/l, primary endpoint) when initiated on day 1 of sensor insertion vs day 3-4 were comparable (58 ± 32% day 1 vs 56 ± 36% day 3-4; *P* = .34), and there were no significant differences between interventions in terms of mean plasma glucose (*P* = .26), percentage time above 8.0 mmol/l (*P* = .49), and time spent below 3.9 mmol/l (*P* = .93). Sensor accuracy varied with sensor life (mean absolute relative difference 19.8 ± 15.0% on day 1 and 13.7 ± 10.2% on day 3 to 4). Sensor glucose tended to under-read plasma glucose inflating benefits of CL on glucose control.

**Conclusions:**

In spite of differences in sensor accuracy, overnight CL glucose control informed by sensor glucose on day 1 or day 3-4 after sensor insertion was comparable. The model predictive controller appears to mitigate against sensor inaccuracies.

The emergence of accurate and easy-to-use continuous glucose monitoring (CGM) devices, which enable users to view in real-time estimates of plasma glucose and receive alarms for impending hypo- or hyperglycemia, is a major step toward improved glucose monitoring and facilitates appropriate changes to insulin therapy. CGM is becoming increasingly popular in people with type 1 diabetes across all age-groups.^1^ Clinical benefits, particularly in combination with insulin pump therapy, are conditioned on good adherence.^2,3^

CGM-assisted therapeutic approaches beyond diabetes monitoring include threshold-suspend and predictive low glucose management insulin pump therapy, which temporarily interrupt insulin delivery at predefined low sensor glucose levels, and thus may alleviate the burden of hypoglycemia.^4,5^ Closed-loop (CL) systems expand on the concept of sensor responsive insulin delivery using a control algorithm that autonomously modulates insulin delivery below and above preset insulin pump delivery based on real-time sensor glucose levels.^6^ CL approaches are increasingly tested in outpatient and home settings.^7-11^

Glucose control during CL application depends to a large extend on the accuracy and reliability of CGM systems. CGM accuracy and reliability has improved due to advances in sensor technology, data processing and calibration algorithms.^12,13^ However, consistent glucose sensor function over the full lifetime of a sensor may be unattainable and sensors are least accurate in the 24-hour period immediately postinsertion compared to half-way through sensor life (say days 3 to 4).^14,15^ This may relate to insertion trauma causing onset of an inflammatory response and tissue microhemorrhage which may resolve with time.^16-18^

The purpose of the present study was to evaluate the effect of sensor life on CL performance comparing CL efficacy and safety on day 1 of sensor insertion to day 3 to 4 of sensor insertion in young people with type 1 diabetes over an overnight period at a clinical research facility. We hypothesized that more accurate sensor performance as usually seen half-way through sensor life, may lead to better CL performance as assessed by frequent plasma glucose measurements.

## Methods

An open label randomized 2-period crossover study compared overnight CL insulin delivery on day 1 of sensor insertion versus day 3 to 4 of sensor insertion. The study was designed as a pilot trial. Prior to study initialization, approval was sought and received from the local independent research ethics committee and the UK regulatory authority (Medicines & Health products Regulatory Agency). Participants aged ≥16 years and parents or guardians of participants aged <16 years signed informed consent; written assent was obtained from minors.

### Subjects and Study Protocol

The study was conducted at the Wellcome Trust Clinical Research Facility at Addenbrooke’s Hospital, Cambridge, between May 2014 and April 2015. Children and adolescents aged 6-18 years were recruited from 3 pediatric diabetes clinics at Cambridge, London University College Hospital, and Peterborough. Eligibility criteria included type 1 diabetes (WHO criteria) for at least 12 months, insulin pump therapy for at least 3 months, glycated hemoglobin (HbA1c) below 97 mmol/mol (11%) based on analysis from local laboratory within 3 months. Exclusion criteria included any physical or psychological disease likely to interfere with the normal conduct of the study and data interpretation or current treatment with drugs likely to interfere with glucose metabolism.

Medtronic MiniMed Paradigm® Veo™ insulin pumps (MMT-554 or MMT-754) with second-generation Enlite™ CGM sensors (Medtronic MiniMed, Northridge, CA, USA) were used as study pump and continuous glucose monitoring systems (CGM). At recruitment, participants received training on real-time CGM component of the Veo system, and participants’ competency in using CGM was assessed and documented by the clinical investigators. No additional pump training was provided as all participants recruited for this trial were already using Veo insulin pump prior to enrolment. CGM calibration followed manufacturer’s instructions using finger-stick glucose measurements taken every 12 h on CONTOUR XT Meter (Bayer, Leverkusen, Germany) which was checked for accuracy by calibration fluid.

**Figure 1.**
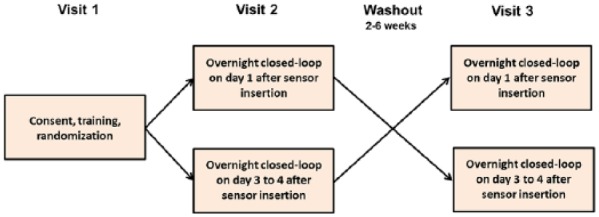
Study design.

Participants attended the clinical research facility for 2 overnight periods, 2 to 6 weeks apart, with identical study protocol performed on both occasions. On 1 occasion, the CL system was informed by a glucose sensor inserted in the morning of the study visit, and on the other occasion study participants had been fitted with a CGM sensor 3 to 4 days prior to the study visit. The order of the interventions was random according to a computer-generated allocation sequence with permuted blocks (Figure 1).

**Figure 2.**
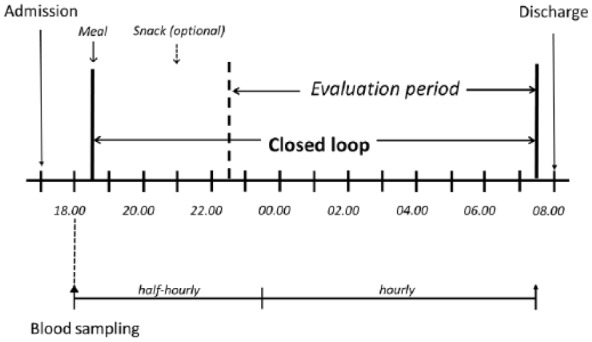
Schematic presentation of overnight study visit schedule. Identical procedures were followed during both study visits.

On each occasion, participants were admitted at 17:00 and stayed until 08:00 the following day (Figure 2). An intravenous cannula was placed for blood sampling starting at 18:00. Participants consumed an evening meal at 18:30 (74 ± 27 g carbohydrates) and an optional bedtime snack at 21:00 (23 ± 15 g carbohydrates). The meals and snacks were identical on the 2 occasions. Meals and carbohydrate content were chosen by the children and their families based on individual preferences and reflecting usual practice at home. Meals were accompanied by insulin boluses calculated using participants’ standard insulin pump bolus calculator settings and premeal finger-stick glucose levels. Rapid acting insulin analogue aspart (Novo Nordisk, Bagsvaerd, Denmark) was used.

### CL System

**Figure 3.**
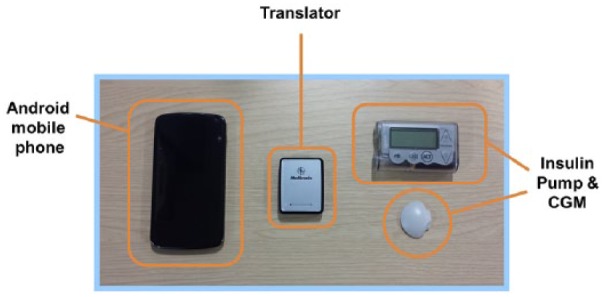
Amber CL platform comprising Android phone running the model predictive control algorithm, translator facilitating wireless communication between the phone and the Veo pump, and CGM transmitter connected to Enlite sensor.

The Amber system Android CL platform employed an Android smartphone (Nexus 4, LG, South Korea) running a model predictive control algorithm (version 0.3.30, University of Cambridge) embedded in user interface module and communicating with a Bluetooth to radiofrequency translator module linked to Veo pump (all Medtronic MiniMed; Figure 3). Every 15 min, the control algorithm automatically initiated a new insulin infusion rate based on sensor glucose through wireless communication. The calculations utilized a compartment model of glucose kinetics describing the effect of rapid-acting insulin analogues and the carbohydrate content of meals on glucose levels. The control algorithm was initialized by downloading preprogrammed basal insulin doses from the pump. In addition, information about participant’s weight and total daily insulin dose were entered at setup. No plasma glucose data were provided to the algorithm.

### Sampling and Assays

For the measurement of glucose and insulin concentration, venous blood samples were obtained every 30 minutes until 23:30, then hourly from 23:30 to 07:30. Plasma was separated by centrifugation immediately. Plasma glucose levels were determined in real time by YSI2300 STAT Plus analyzer (Yellow Springs Instrument, Farnborough, UK) but were not used to inform the algorithm. Plasma insulin concentration was measured by immunochemiluminometric assay (IV2-001; Invitron Ltd, Monmouth UK) with an interassay variation of 7.1%, 2.4% and 7.1% at 89 pmol/L, 488 pmol/L, and 873 pmol/L, and an analytical sensitivity of 0.12 pmol/L.

### Study Outcomes

The primary outcome was the time during CL when plasma glucose levels were within the target range from 3.9 to 8.0 mmol/l in the overnight period from 22:30 until 07:30 on the following day.

Secondary outcomes included mean plasma glucose levels, glucose variability, time spent below and above the target range during observation period (22:30 to 07:30). All glucose outcomes were also compared with CGM sensor values. Glucose variability was assessed by the standard deviation and the coefficient of variation. Hypoglycemia burden was assessed by calculating the glucose area under the curve less than 3.5 mmol/l. Insulin delivery amounts were reported as total overnight basal insulin delivery. Sensor accuracy for each study arm was assessed using paired sensor and plasma glucose points. The bias (sensor minus plasma value) and relative absolute difference (RAD) (absolute difference divided by the reference value, expressed as percentage) were computed for each pair. Numerical accuracy outcomes were calculated across the whole range of measured glucose levels, as well as for euglycemic (3.9-10.0 mmol/L), hypoglycemic (<3.9 mmol/L), and hyperglycemic (>10.0 mmol/L) ranges stratified according to plasma glucose measurements. Clinical accuracy was assessed by Clarke error grid analysis.

### Statistical Analysis

The statistical analysis plan was agreed on by investigators in advance. All analyses were undertaken on an intention-to-treat basis. The respective values obtained during the 2 overnight randomized interventions were compared using a least-square regression model. Glucose outcomes and insulin outcomes were adjusted for period effect. Rank normal transformation analyses were used for highly skewed endpoints. Outcomes were presented as mean ± SD for normally distributed values or as median (interquartile range) for non-normally distributed values. Accuracy outcomes were summarized using descriptive statistics. Efficacy and accuracy metrics were calculated by GStat software (University of Cambridge, version 2.2), and statistical tests were carried out using SPSS software (IBM Software, Hampshire, UK version 21). A 5% significance level was used to declare statistical significance for all comparisons. All *P* values are 2-sided.

## Results

### Participants

We approached 15 patients with type 1 diabetes, of whom 13 participants consented. One participant dropped out during run-in (loss to follow-up). In all, 12 subjects were randomized, completed both study periods, and provided data for analyses (Table 1).

**Table 1.** Baseline Characteristics of Study Participants.

Subjects (n)	12
Female (n)	3
Age (years)	16.7 ± 1.9
Weight (kg)	68.6 ± 16.8
BMI (kg/m^2^)	21.6 ± 3.3
BMI *z*-score	0.26 ± 1.26
Duration of diabetes (years)	8.7 ± 3.6
Duration of pump use (years)	6.0 ± 2.7
Total daily insulin dose (U/kg/day)	0.90 ± 0.29
Glycated hemoglobin (%)	8.3 ± 1.1
Glycated hemoglobin (mmol/mol)	66 ± 10

Values are mean ± SD, unless otherwise noted.

### Overnight Glucose Control

**Figure 4.**
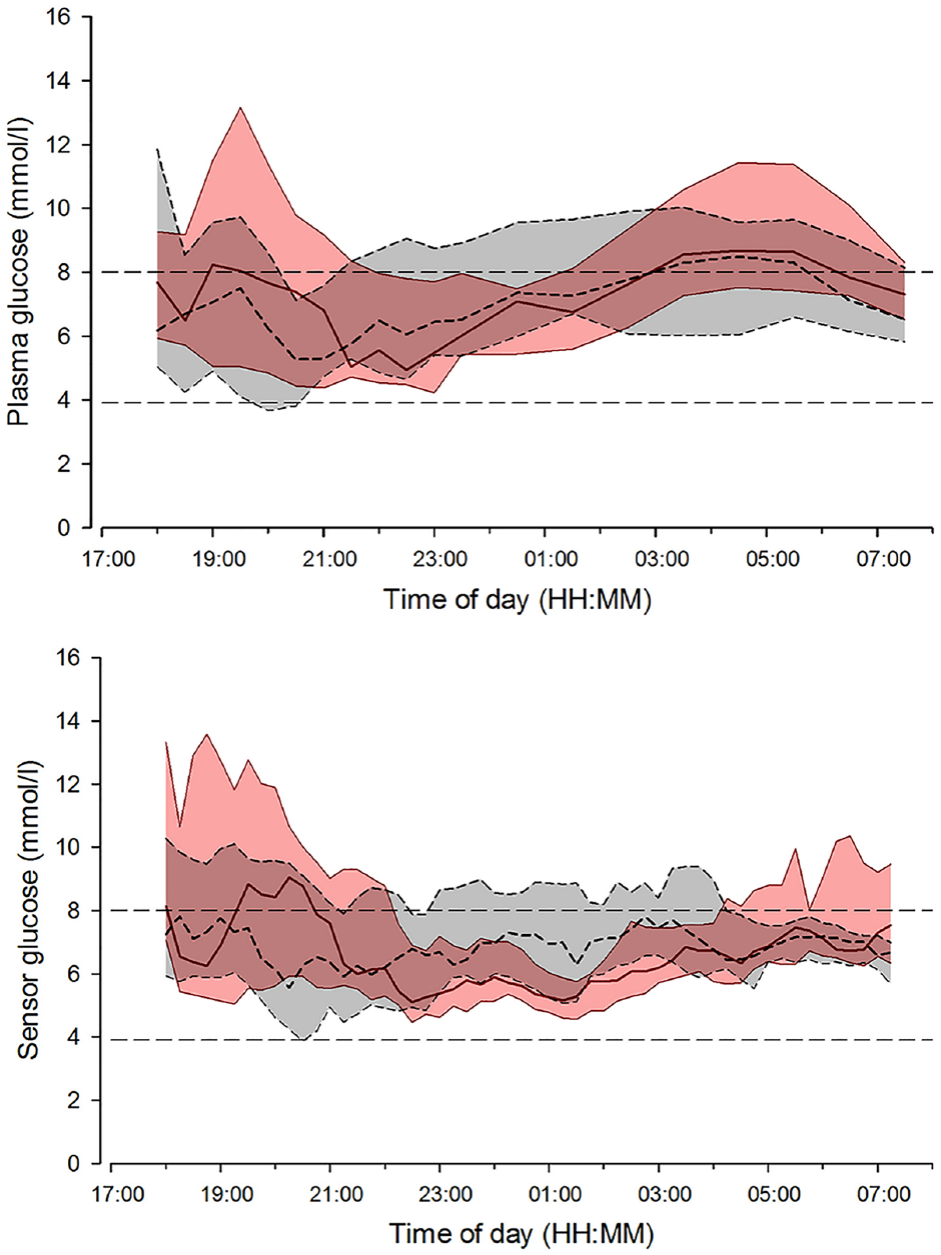
Median (interquartile range) of plasma glucose (top panel) and sensor glucose (bottom panel) during overnight closed loop on day 1 of sensor insertion (solid red line and red shaded area) and closed loop on day 3 to 4 of sensor insertion period (dashed black line and gray shaded area). The glucose range 3.9 to 8.0 mmol/l is denoted by horizontal dashed lines.

Study outcomes during the overnight CL periods (22:30 to 07:30) are summarized in Table 2. Plasma glucose and sensor glucose profiles during CL on day 1 and day 3 to 4 after sensor insertion are shown in Figure 4.

**Table 2.** Comparison of Outcomes During Overnight (22:30 to 07:30) Closed Loop (CL) on Day 1 of Sensor Insertion vs Day 3 to 4 of Sensor Insertion Based on Plasma Glucose and Sensor Glucose Readings.

	Plasma glucose				Sensor glucose				Plasma vs sensor	
	CL day 1 (n = 12)	CL day 3/4 (n = 12)	Paired difference^a^ (95% CI)	*P* value	CL day 1 (n = 12)	CL day 3/4 (n = 12)	Paired difference^a^ (95% CI)	*P* value	Paired difference^a^ (95% CI)	*P* value
Time in target										
3.9-8.0 mmol/l (%)^b^	58 ± 32	56 ± 36	2 (−21 to 24)	.30	73 ± 25	71 ± 21	1 (−19 to 22)	.72	−15 (−27 to −3)	.014
3.9-10.0 mmol/l (%)	87 (63 to 100)	92 (72 to 99)	0 (−12 to 9)	.30	90 (79 to 100)	92 (84 to 98)	2 (−7 to 8)	.33	0 (−7 to 2)	.34
Mean glucose (mmol/l)	7.9 ± 1.6	7.8 ± 1.8	0.1 (−0.9 to 1.0)	.26	6.8 ± 1.4	7.1 ± 0.9	−0.4 (−1.4 to 0.7)	.65	0.9 (0.2 to 1.5)	.010
Hypoglycemia										
Less than 3.9 mmol/l (%)	0.0 (0.0 to 3.2)	0.0 (0.0 to 1.8)	0.0 (−0.4 to 2.6)	.93	1.0 (0.0 to 4.4)	0.0 (0.0 to 3.5)	0.0 (−1.7 to 1.9)	0.41	0.0 (−2.2 to 0.0)	.30
AUC_day_ <3.5 mmol/l (mmol/l x min)	0.0 (0.0 to 5.1)	0.0 (0.0 to 0.0)	0.0 (0.0 to 5.1)	.11	0.0 (0.0 to 3.6)	0.0 (0.0 to 0.0)	0.0 (0.0 to 3.5)	0.37	0.0 (0.0 to 0.0)	.96
Hyperglycemia										
Time spent at glucose levels (%)										
>8.0 mmol/l	39 ± 33	43 ± 36	−4 (−24 to 17)	.49	21 ± 23	26 ± 22	−5 (−23 to 13)	0.78	18 (5 to 30)	.005
>10.0 mmol/l	0 (0 to 37)	9 (0 to 27)	0 (−9 to 1)	.58	1 (0 to 15)	4 (0 to 12)	2 (−7 to 8)	0.68	0 (−1 to 6)	.23
Glucose variability										
SD of glucose (mmol/l)	1.2 (1.2 to 2.1)	1.4 (1.0 to 2.0)	0.0 (−0.5 to 0.8)	.26	1.5 (0.8 to 2.3)	1.4 (0.5 to 1.4)	0.0 (−0.3 to 0.5)	0.29	0.1 (−0.2 to 0.2)	.92
CV of glucose (%)	18 (15 to 28)	18 (14 to 23)	0 (−5 to 10)	.38	22 (13 to 34)	20 (14 to 25)	2 (−7 to 12)	0.15	−1 (−4 to 2)	.63

Values are mean ± SD or median (interquartile range).

a Normally distributed data are presented as mean difference of values (CL day 1 minus CL day 3 to 4, or plasma glucose minus sensor glucose), with 95% confidence interval for the mean. Non-normally distributed data are presented as median difference of values (CL day 1 minus CL day 3 to 4, or plasma glucose minus sensor glucose), with 95% confidence interval for the median.

b Primary endpoint.

### Plasma Glucose Outcomes

Plasma glucose levels remained within the target range of 3.9 to 8.0 mmol/l (primary endpoint) for 58% and 56% of the time, respectively, when CL was applied on day 1 or on day 3 to 4 (*P* = .30, Table 2). No difference was found in the mean plasma glucose concentration (7.9 ± 1.6 vs 7.8 ± 1.8 mmol/l, *P* = .26). Proportion of time when plasma glucose was in hypoglycemic range (below 3.9 mmol/l) and the area under the curve when plasma glucose was less than 3.5 mmol/l were very low and comparable during the study periods. There was no difference in glucose variability between study periods as measured by the standard deviation and coefficient of variation of plasma glucose.

### Sensor Glucose Outcomes

The proportion of time that sensor glucose was in the target glucose range 3.9 to 8.0 mmol/l during CL was comparable on day 1 and day 3 to 4 of sensor use (73 ± 25% vs 71 ± 21%, *P* = .72; Table 2). Similarly, there was no difference in mean sensor glucose levels, the proportion of time above and below the target range, and variability in glucose readings during the 2 overnight study periods (Table 2).

### Plasma vs Sensor Glucose

Despite similar CL outcomes between interventions, direct comparison of sensor glucose readings and plasma glucose derived indices showed marked differences (Table 2). The proportion of time spent in target range 3.9 to 8.0 mmol/l was significantly higher when calculations were based on sensor readings than plasma glucose based calculations (*P* = .014). Mean sensor glucose levels were significantly lower than mean plasma glucose readings (*P* = .010) as was time spent above target range (*P* = .005).

### Insulin Delivery

**Figure 5.**
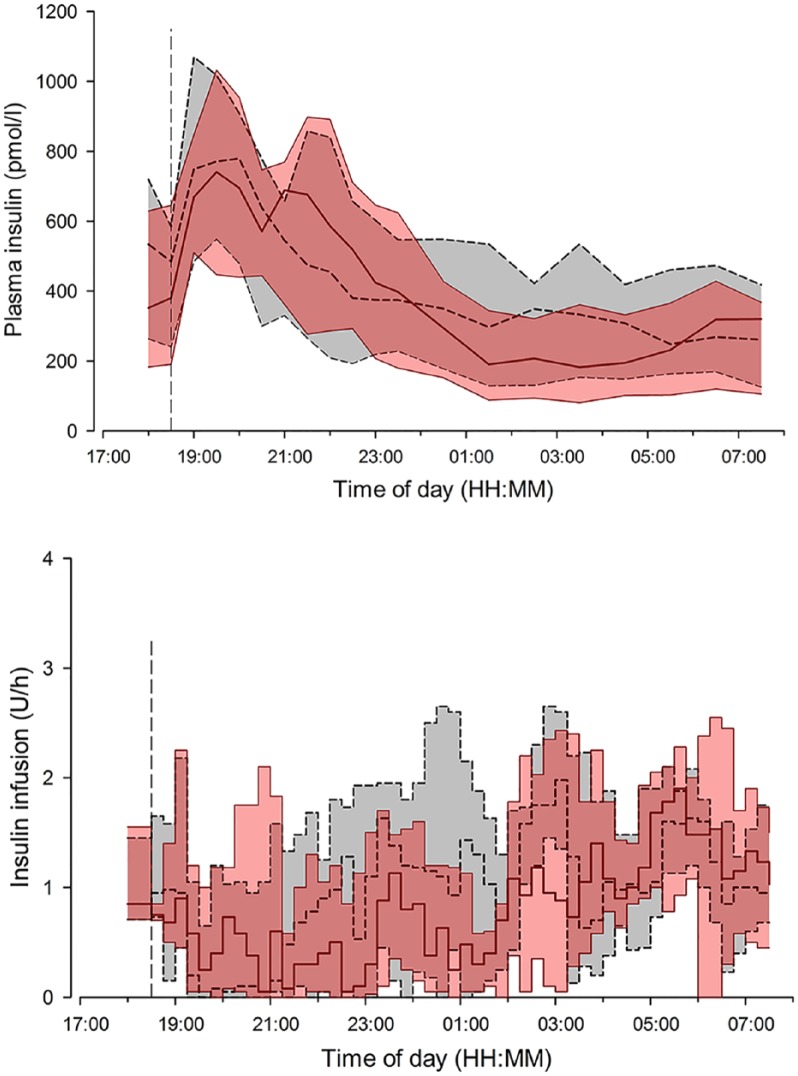
Insulin infusion rates (top panel), and plasma insulin (bottom panel) are shown for closed loop on day 1 of sensor insertion (solid red line and red shaded area) and closed loop on day 3 to 4 of sensor insertion (black dashed line and gray shaded area [interquartile range]). The vertical dashed line indicates when closed loop started and the evening meal was consumed.

Total overnight insulin delivery (22:30 to 07:30) did not differ between interventions (8.4 [6.0 to 14.4]U on day 1 of sensor insertion vs 11.3 [8.8 to 15.3]U on day 3 to 4, of sensor insertion vs 11.3 [8.8 to 15.3]U on day 3 to 4, *P* = .13), and resulted in similar plasma insulin levels (Figure 5). Variability in insulin delivery was similar during the 2 overnight visits (SD 0.7 [0.6 to 1.0]U vs 0.8 [0.6 to 0.9]U, *P* = .84).

### Sensor Accuracy

**Figure 6.**
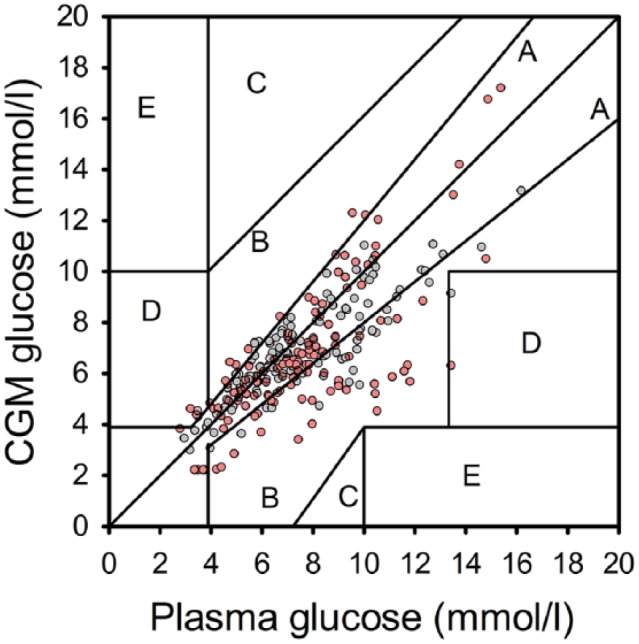
Clarke error grid of sensor and plasma glucose levels shown for CL on day 1 of sensor insertion (red dots) and CL on day 3 to 4 of sensor insertion (gray dots). Data collected from 22:30 to 07:30 are presented.

Sensor accuracy evaluation is summarized in Table 3. On the CL nights between 22:30 and 07:30, 126 sensor–plasma glucose pairs were analyzed on day 1 after sensor insertion, and 123 on day 3 to 4. Across the whole glucose range, numerical sensor accuracy expressed as mean ARD was 19.8 ± 15.0% on day 1 and 13.7 ± 10.2% on day 3 to 4, respectively (separate sensor performance matrices for euglycemic, hypo-and hyperglycemic ranges are shown in Table 3). Mean bias for sensors on day 1 was -0.9 ± 1.9 mmol/l, and -0.7 ± 1.4 mmol/l on day 3 to 4, respectively. On day 1 of insertion the new generation Enlite sensor had 96.8% of measurements in Clarke error grid zones A+B (zone A, 61.9%; zone B, 34.9%; zone C, 0%; zone D, 3.2%; zone E, 0%; Table 3 and Figure 6). On day 3 to 4, 98.3% of paired sensor data points were in zones A+B (zone A, 71.5%; zone B, 26.8%; zone C, 0%; zone D, 1.6%; zone E, 0%) (Table 3 and Figure 6).

**Table 3.** Numerical and Clinical Accuracy on Day 1 Compared to Day 3 to 4 After Sensor Insertion (Evaluated From 22:30 to 07:30).

	CL on day 1 of sensor insertion (n = 12)	CL on day 3 to 4 of sensor insertion (n = 12)
Number of paired points	126	123
Mean plasma glucose (mmol/l)	7.9 ± 1.6	7.8 ± 1.8
Clarke error grid (%)		
Zone A	61.9	71.5
Zone B	34.9	26.8
Zone C	0	0
Zone D	3.2	1.6
Zone E	0	0
Median bias (mmol/l)	−0.6 (−1.6 to 0.4)	−0.2 (−1.7 to 0.3)
Mean bias (mmol/l)	−0.9 ± 1.9	−0.7 ± 1.4
Whole range (2.2-17.9 mmol/l)		
Median AD (mmol/l)	1.1 (0.5 to 1.8)	0.8 (0.3 to 1.7)
Median ARD (%)	16.3 (7.5 to 28.6)	12.6 (4.7 to 20.9)
Mean AD (mmol/l)	1.5 ± 1.5	1.1 ± 1.0
Mean ARD (%)	19.8 ± 15.0	13.7 ± 10.2
Euglycemia (3.9-8.0 mmol/l)		
Number of paired points	71 (56%)	64 (52%)
Median ARD (%)	12.9 (7.5 to 28.5)	8.5 (4.2 to 16.9)
Hypoglycemia (<3.9 mmol/l)		
Number of paired points	8 (6%)	6 (5%)
Median ARD (%)	37.0 (31.0 to 40.2)	8.5 (5.0 to 17.8)
Hyperglycemia (>8.0 mmol/l)		
Number of paired points	47 (37%)	64 (43%)
Median ARD (%)	20.0 (7.4 to 37.4)	18.6 (7.0 to 25.2)

AD, absolute difference; ARD, absolute relative difference; bias, plasma glucose minus sensor glucose.

Values are mean ± SD or median (interquartile range).

## Discussion

We document that overnight CL glucose control using a model predictive control algorithm informed by sensor glucose on day 1 after insertion was similar to that achieved when CL was initiated on day 3 to 4 of sensor life. Glucose levels were maintained between 3.9 and 8.0 mmol/l for a similar proportion of time, mean plasma glucose readings were comparable, and there were no differences in hypoglycemia burden.

We observed sensor accuracy comparable to previously reported data,^14,15,19-21^ including reduced accuracy on day 1 of sensor insertion.^14,15,21^ Notwithstanding the reduced accuracy on day 1, sensor life did not affect performance of our CL system. We hypothesized that this is related to the robustness of our model predictive algorithm, which mitigates against sensor inaccuracy and has been safely and effectively used in a range of populations and settings including pregnant women, adults, adolescents and children in unsupervised home application.^10,22,23,24^ The relatively slow kinetics of subcutaneous insulin may inherently increase robustness of CL algorithms as short-lived aberrations in sensor glucose on day 1, translating to short-term deviations from ideal insulin delivery, may have limited impact on glucose control as these insulin delivery errors cancel each other. However, prolonged sensor inaccuracies cannot be accommodated in this fashion.

Outcomes based on sensor readings significantly inflated CL performance compared to plasma glucose outcomes. This is in contrast to findings from an overnight inpatient study with a similar design conducted in young children using another sensor make, when plasma and sensor based outcomes were comparable.^25^ In the present study, sensor reported glucose levels significantly lower than plasma glucose values. A similar trend was described by Calhoun et al.^15^ In view of these results, previously reported glucose outcomes based on Enlite sensor, including to benefits of sensor-based therapy regimen (eg, sensor-augmented pump therapy, low glucose suspension, CL trials), should be interpreted with caution.

The current study was limited by the relatively small sample size and short overnight intervention periods. A limited number of data points for sensor accuracy assessment were collected, particularly with respect to the hypoglycemic range. The strengths of our study included the crossover randomized design, and the controlled environment to exclude potential confounders with respect to this particular research question.

In conclusion, overnight CL glucose control in adolescents informed by Enlite glucose sensor on day 1 or day 3 to 4 after sensor insertion was comparable. The model predictive controller appears to mitigate against sensor inaccuracies.
